# Supplementary material for: How does priority setting for resource allocation happen in commissioning dental services in a nationally led, regionally delivered system: a qualitative study using semistructured interviews with NHS England dental commissioners
Source: BMJ Open. 2019 Mar 23;9(3):e024995. doi: 10.1136/bmjopen-2018-024995 (PMC6475363; doi:10.1136/bmjopen-2018-024995)
Supplement: Supplementary file 1 [file bmjopen-2018-024995supp001.pdf]

**RAINDROP: Resource Allocation in NHS Dentistry: Recognition of Societal Preferences**
**Topic Guide**

N.B. Key questions are in normal type. *Prompts to be used if necessary are in italics*

Can you tell me a bit about your previous roles relevant to commissioning?
*Previous jobs and positions in NHS administration*
*Dental experience*
*Change of roles with change of NHS configurations in 2012*

Can you outline what you do in your current role as a dental commissioner?
*Day to day work*
*Key relationships/structures*
*Strategic/specific projects involved with currently*

How would you describe the relationships in commissioning currently?
*With other commissioners inside/outside (CCGs) of dentistry*
*With dentists (LPNs)*
*With providers – primary (LDCs)/secondary care*
*With Dental Public Health*
*With Local Authorities*
*With HEE*
*With patients/healthwatch*

Thinking about dental commissioning nationally, what things work well?
*Relationships/Structures/Processes/Support/Leadership*

Thinking about dental commissioning nationally, what would you say are some of the aspects that are most frustrating or most need to change?
*Relationships/Structures/Processes/Support/Leadership*

What is the relationship and split of responsibilities between the national/central team and locally?

What do you currently feel about the balance of contracting versus commissioning in your personal role and nationally?

What would need to change to allow a move from contracting to commissioning?
*Relationships*
*Structures*
*Processes*
*Support*
*Leadership*

In your commissioning decisions, what ways do you set priorities for investment/projects?
*Do you invest/disinvest?*
*What criteria do you judge a new scheme on?*
